# Randomised control study of remote ischaemic preconditioning and hepatic function

**DOI:** 10.1186/1749-8090-8-S1-P71

**Published:** 2013-09-11

**Authors:** A Khan, A Bose

**Affiliations:** 1 Dept. of Cardiothoracic Surgery, Victoria Hospital, Blackpool, UK

## Background

Remote Ischemic preconditioning is a technique whereby ischemia is induced in a remote non vital tissue based on the hypothesis that this initial ischemia enhances the tolerance of other organs to cope with subsequent prolonged ischemia. The effect of remote Ischemic preconditioning and hepatic function is less studied.

## Methods

After the induction of general anesthesia, patients are randomized either to receive the intervention or not(control). Those who are randomized to receive intervention have 4 cycles of 5 min inflation to 200 mmHg and 5 min deflation of a blood pressure cuff placed on the upper arm. The control patients have 4 cycles of simulated inflations and deflations of the blood pressure cuff. Hepatic function was studied using Serum Bilirubin, Serum Alkaline Phosphatase and (Adjusted Calcium). These parameters were recorded pre-operatively and post-operatively at day 2. The secondary end point was 30 day mortality. The aim was to see if Remote Ischemic preconditioning is protective against liver injury. (Trial Identifier: NCT01247545).

## Results

We present the preliminary findings of our Remote Ischemic preconditioning randomized control trial. We look at the first 23 patients who were enrolled in the trial. 10 Patients were randomized in the intervention group and 13 patients were in the control group. The mean Additive EuroSCORE for the Intervention group was 6.33 ± 1.8 and for the control group was 6.9 ± 2.0. The initial results show no statistical difference in the hepatic function between the Intervention group and control group.

## Conclusion

The initial results show no statistical difference in the liver function between the Remote Ischemic preconditioning group and the control group. This is a proof of concept study with early results showing no statistical difference. We look forward to analyzing the full results.

